# cGMP-dependent kinase 2, Na^+^/H^+^ regulatory factor 2, and Na^+^/H^+^ exchanger isoform 3 assemble within lipid rafts in murine small intestinal brush border membrane

**DOI:** 10.1186/2050-6511-14-S1-O31

**Published:** 2013-08-29

**Authors:** Min Luo, Ayesha Sultan, Qin Yu, Brigitte Riederer, Weiliang Xia, Min Luo, Mingmin Chen, Simone Lissner, Engelbert Gessner, Enrico Patrucco, Franz Hofmann, Mark Donowitz, Chris C Yun, Hugo de Jonge, Georg Lamprecht, Ursula Seidler

**Affiliations:** 1 Dept. of Gastroenterology, Hepatology and Endocrinology, Hannover Medical School, Germany; 2 Dept. of Gastroenterology, Tongji Hospital, Huazhong University of Science and Technology, China; 3 Key Lab of Combined Multiorgan Transplantation, 1st Affiliated Hospital, School of Medicine, Zhejiang University, Hangzhou, China; 4 1st Medical Department, University of Tübingen, Tübingen, Germany; 5 Dept. of Immunology and Rheumatology, Hannover Medical School, Germany; 6 Forschergruppe 923, Institut für Pharmakologie und Toxikologie, TU München, Biedersteiner Str. 29, 80802, München, Germany; 7 Division of Gastroenterology, Department of Medicine and Physiology, John Hopkins School of Medicine, Baltimore, USA; 8 Division of Gastroenterology, Dept. of Medicine, Emory University, Atlanta, USA; 9 Dept. of Gastroenterology, Erasmus MC, Rotterdam, The Netherlands; 10 Dept. of Gastroenterology, University of Rostock, Germany

## 

Trafficking, brush border membrane (BBM) retention, and signal-specific regulation of the Na^+^/H^+^ exchanger NHE3 is regulated by Na^+^/H^+^ Exchanger Regulatory Factor (NHERF) family of PDZ-adapter proteins, which enable the formation of multiprotein complexes. It is unclear, however, what determines signal specificity of the very homologous NHERFs. We studied the association of NHE3, as well as NHERF1 (EBP50), NHERF2 (E3KARP) and NHERF3 (PDZK1) with lipid rafts in murine small intestinal BBM and their possible association with signaling molecules. NHE3 was found to partially associate with glycosphingolipid-enriched microdomains in the native BBM, and NHE3 raft association had an impact on NHE3 transport activity as well as on second-messenger-dependent regulation *in vivo*. NHERF1, 2 and 3 were differentially distributed to rafts and non-rafts, with NHERF2 being most raft-associated and NHERF3 entirely non-raft associated. A search for other signalling molecules that are implicated in regulating NHE3 through a NHERF interaction, cGMP-dependent kinase II, which together with NHERF2 is essential for guanylin/heat stable enterotoxin of E.coli (STa)-mediated NHE3 inhibition in the intestine, was found exclusively lipid-raft associated. In conclusion, the differential association of the NHERFs, as well as kinases, with the raft-associated and the non-raft fraction of NHE3 in the brush border membrane is likely one component of the differential and signal-specific NHE3 regulation by the different NHERFs.

